# Water transport proteins–aquaporins (AQPs) in cancer biology

**DOI:** 10.18632/oncotarget.26351

**Published:** 2018-11-20

**Authors:** Salah Dajani, Anand Saripalli, Neelam Sharma-Walia

**Affiliations:** ^1^ H.M. Bligh Cancer Research Laboratories, Department of Microbiology and Immunology, Chicago Medical School, Rosalind Franklin University of Medicine and Science, North Chicago, Illinois, USA

**Keywords:** aquaporins, breast cancer, reactive oxygen species, antioxidant, inflammation

## Abstract

As highly conserved ubiquitous proteins, aquaporins (AQPs) play an imperative role in the development and progression of cancer. By trafficking water and other small molecules, AQPs play a vital role in preserving the cellular environment. Due to their critical role in cell stability and integrity, it would make sense that AQPs are involved in cancer progression. When AQPs alter the cellular environment, there may be several downstream effects such as alterations in cellular osmolality, volume, ionic composition, and signaling pathways. Changes in the intracellular levels of certain molecules serving as second messengers are synchronized by AQPs. Thus AQPs regulate numerous downstream effector signaling molecules that promote cancer development and progression. In numerous cancer types, AQP expression has shown a correlation with tumor stage and prognosis. Furthermore, AQPs assist in angiogenic and oxidative stress related damaging processes critical for cancer progression. This indicates that AQP proteins may be a viable therapeutic target or biomarker of cancer prognosis.

## INTRODUCTION

**Figure 1:**
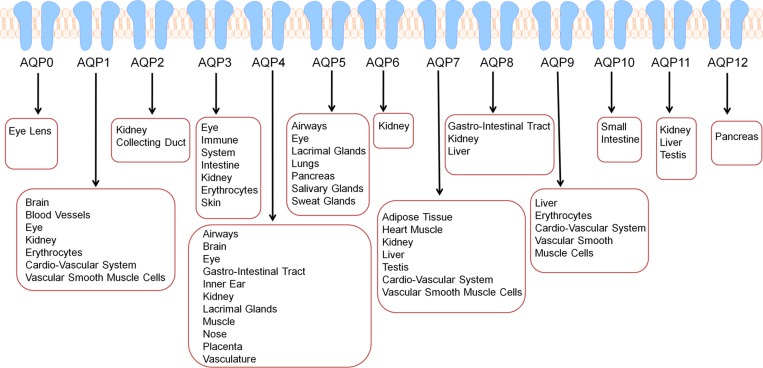
AQPs are expressed in dozens of tissue types in humans further reinforcing that altered AQP expression may have implications in numerous cancer types in various sites Besides their presence and functionalty in tissues, AQPs are also circulating in exosomes or extracellular vesicles and are also expressed in human erythrocytes.

Aquaporins (AQPs) play a pivotal role in life and are responsible for maintaining water homeostasis and solute transfer. AQPs are small integral membrane water transport proteins that allow water to flow through cell membranes in response to osmotic gradients in cells. The AQP family in mammals consists of thirteen unique members denoted AQP0-AQP12 [1]. AQPs belong to a highly conserved group of membrane-spanning proteins called the major intrinsic proteins that are found in practically all living organisms. Additionally, AQPs are found in dozens of different tissue types throughout the body (Figure 1). Human erythrocytes express large amounts of AQP1, AQP3 and AQP9 (Figure 1), which play roles in bidirectional transfer of water and small solutes across cell membranes [2]. AQP3, AQP7, AQP9, and AQP10 are also called aquaglyceroporins since they are permeable to both glycerol and water. Activity and expression of various AQPs is usually analyzed to determine the effectiveness of new drugs, assessing membrane permeability and regulation of energy homeostatsis [2].

**Figure 2:**
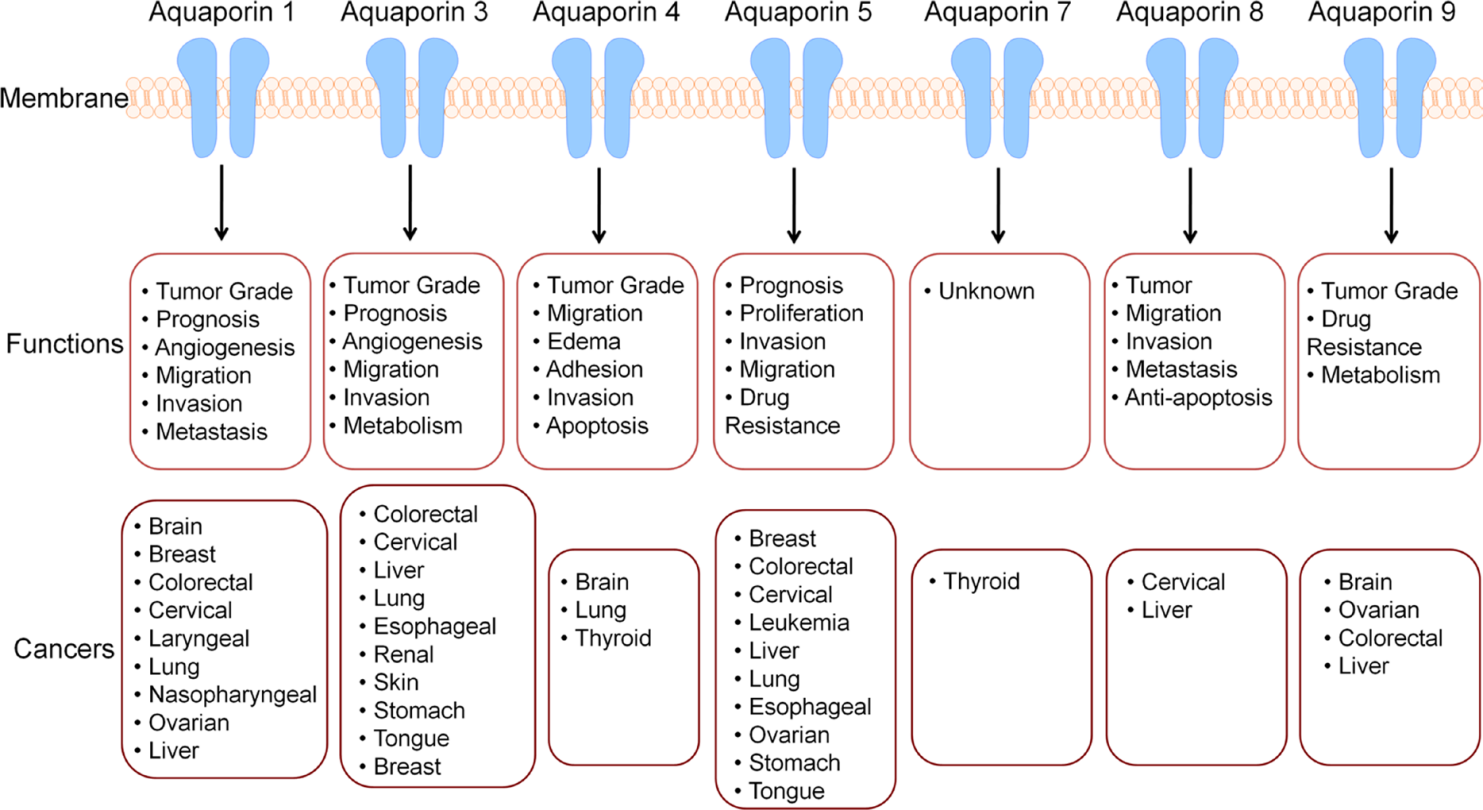
AQPs display altered expression various cancer types and are implicated in numerous processes Since cancer is a disease of failing molecular machinery, it is important to note that these malfunctions can occur in any region where the molecular machinery is normally located. There is relationship between various cancer types and AQP channels. AQPs are implicated in numerous cancer types and various processes, abolishing the previous belief that they play a passive role in only water transport. Alterations in AQP expression indicate AQPs may be used as a biomarker, prognostic factor, and therapeutic target.

AQPs consist of monomers that are approximately 30kDa, possessing 6 transmembrane alpha-helical domains. When AQP channels assemble, the monomers merge to form homotetramers, leading to four linked transport channels [3]. Typically, AQPs are divided into two families based on transfer specificity, classical water transporting AQPs and solute transporting aquaglyceroporins. However, AQPs also play a part in the transport of ammonia, urea, carbon dioxide, metalloids, nitric oxide, and some ions [2]. Although AQPs allow the transport of various substrates, they tend to restrict charged substrates such as cations, protons, inorganic cations, and ammonium. However, AQPs do tend to show permeability for anions [4]. Various conditions such as phosphorylation, pH, pressure, temperature, solute gradients, and more, play a role in the gating process of AQPs. A thorough examination of AQPs shows that each AQP type plays a meaningful role such as facilitating migration, invasion, metastasis, proliferation, drug resistance, and subsequent prognosis in specific cancer type(s) (Figure 2). Two regions of the channel are critical for AQP function. These regions are Asn-Pro-Ala or NPA and the aromatic-arginine region [5]. The NPA region is in the middle of the channel. It participates in proton exclusion and assists to localize AQPs to the plasma membrane. The aromatic-arginine region is towards the extracellular side and acts as a selectivity filter, blocking particles larger than water [5]. Due to the substantial role of AQPs and their ubiquitous presence, manipulation of AQP function may serve a beneficial therapeutic role. Short-term regulation of AQPs, known as gating, is typically achieved through protein channel conformational changes that impact transport activity [2]. Transport activity of AQPs after the use of inhibitors or cell environment modifications may be monitored through various permeability measurements obtained through the use of epithelial cell permeability assays, osmotic swelling assays, microscopy, stopped-flow spectroscopy, or computational methods. Careful AQP functional analysis is necessary to determine the effectiveness of inhibitors in desired tissues. Recently it has also been discovered that AQPs serve a vital function in simple organisms such as yeast by acting as an oxygen transporter [6]. Furthermore, AQPs directly play a significant role in at least three of the well-known hallmarks of cancer. AQPs are able to contribute to angiogenic processes, which are pivotal for supplying nutrients and substances necessary for tumor progression, and AQPs are implicated in tumor invasion and metastatic potential. AQPs also facilitate and enhance the transport of reactive oxygen species (ROS) and aggravate carcinogenesis and tumor progression.

## AQUAPORINS IN VARIOUS CANCERS

### Breast cancer

There is an increased expression of AQP1, AQP3, and AQP5 in breast cancer (Figure 2) [7]. AQP1 induces the development of angiogenesis by stimulating endothelial cells via estrogen receptors. Furthermore, estrogen may induce AQP1 expression by activating estrogen response element in the promoter of AQP1 gene [7]. AQP1 expression shows a correlative relationship with hypoxia-inducible factor 1 (HIF1). In a study where breast cancer cases were divided into two groups, HIF1-positive and HIF1-negative, in the HIF1-positive groups, the expression level of AQP1 was significantly higher than it was in the HIF1-negative group [8]. Based on the correlations between AQP1 and HIF1 it is assumed that these proteins interact with each other and may regulate the oncogenesis of breast cancer (Figure 2). Hypoxia is a common feature in solid tumors and contributes to the development of malignant tissues and metastasis. Therefore, understanding AQP1 and HIF1 relationships is useful for both therapeutic and prognostic purposes. In another study using AQP1 deficient mice, both breast and lung cancer progression was evaluated. Tumor mass, vessel density, and lung metastasis were found to be lower when compared to mice with normal AQP1 levels [9]. This altered tumor progression indicates that endothelial cell migration is blocked, preventing tumor angiogenesis (Figure 2). AQP3 plays a critical role in the migration of human breast cancer cells induced by fibroblast growth factor-2 (FGF-2) [10]. AQP3 has been shown to control breast cancer cell migration via regulation of hydrogen peroxide (H_2_O_2_) transport, influencing downstream signaling [11]. AQP3 also serves to encourage cell migration and invasion in estrogen receptor positive breast cancer by influencing expression of molecules critical to epithelial mesenchymal transition (EMT) and reorganization of the actin-cytoskeleton (Figure 2) [12]. AQP3 expression is also stimulated by 5′-deoxy-5-fluoropyrimidine nucleosides, which are used in chemotherapy of solid tumors. This may interfere with the effectiveness of chemotherapy; therefore, AQP3 may be a viable therapeutic target to increase the efficacy of chemotherapy. Additionally, in the mammary gland AQP3 may play a role in allowing the transport of glycerol into the cell, thus promoting the availability of intracellular ATP, fueling the demands of growth. Therefore, inhibition of AQP3 may slow proliferation [13]. AQP5 displays increased expression in invasive ductal carcinoma [7]. AQP5 is understood to regulate proliferation and migration of breast cancer cells and may be used as a prognostic marker (Figure 2) [7]. The overexpression of AQP3 and AQP5 may serve as a novel therapeutic marker in patients with triple-negative breast cancer (TNBC) (Figure 2) [14].

### Neuronal cancers

AQP1 and AQP4 are upregulated in high-grade astrocytomas in comparison to low-grade tumors or normal brain tissue (Figure 2) [15, 16]. AQP1, AQP4, and AQP9 are markedly overexpressed in neurological tumors. AQP1 overexpression is typically found in perivascular regions of the brain, indicating its role in angiogenesis [7]. AQP4 has been demonstrated as a significant factor in glioma malignancies regulating invasion, migration of gliomas and edema formation (Figure 2) [17]. By altering H_2_O_2_ transport, AQP4 promotes the development of brain edema formation and glioblastoma migration (Figure 2) [7].

### Pulmonary cancers

AQP1 is upregulated in lung adenocarcinoma, and, inhibition of AQP1 inhibits tumor cell invasion. In lung cancer AQP1 plays a role in promoting angiogenesis and the expression levels of AQP1 was correlated with high postoperative metastasis and low disease-free survival rates [7]. In lung cancer it is common to see overexpression of AQP1, AQP3, AQP4, and AQP5 (Figure 2) [7]. In non-small cell lung cancer tissues, AQP5 has numerous connections to cancer progression. AQP5 shows significantly higher levels of expression in adenocarcinomas in comparison to squamous cell carcinomas. AQP5 in tissues with lymph node metastasis showed higher levels of expression than tissues without lymph node metastasis. AQP5 also showed a positive correlation with the tumor-node-metastasis staging of non-small cell lung cancer. Tissues staged at levels III and IV showed higher levels of AQP5 expression than tissues staged at levels I and II, further indicating the clear role of AQP5 in cancer progression [18]. AQP5 is highly expressed in the respiratory system and secretory glands where it facilitates the osmotically driven generation of pulmonary secretions, saliva, sweat and tears. Dysfunctional trafficking of AQP5 has been implicated in several human disease states impacting pulmonary function such as Sjögren’s syndrome, bronchitis and cystic fibrosis [19].

### Gastrointestinal cancers

AQP1, AQP3, AQP5, and AQP9 all have roles associated with colorectal cancer. Gastric Cancer tissues express higher levels of AQP3 compared to normal gastric mucosa [20, 21]. AQP3 regulated epithelial mesenchymal transition (EMT) associated proteins predicts poor outcome for gastric cancer [22]. AQP3 regulates gastric cancer cell proliferation, invasion and migration through the PI3K/AKT/SNAIL signaling pathway *in vitro* [22]. AQP5 and AQP9 have been associated with drug resistance in colorectal chemotherapy (Figure 2) [7]. AQP1, AQP3, AQP5, AQP8 and AQP9 play a clinically relevant role in hepatic cancer as they do in colorectal cancer (Figure 2). AQP3 promotes stem-like properties of human gastric carcinoma cells by activating the Wnt/GSK-3β/β-catenin signaling pathway. Elevated AQP3 expression was also associated with CD44 expression and activation of the β-catenin signaling pathway in human gastric carcinoma specimens. Altering the AQP3 expression had pronounced effects on the tumorigenic potential and self-renewal capacity of the gastriccancer cells [23].

### AQPs with overlapping oncogenic roles in multiple cancers

In normal epithelial and stromal tissue, there is minimal variation in AQP expression. However, when comparing epithelial and stromal tissue tumors, stromal tumors display significantly higher expression of AQP1, AQP4, AQP6, AQP8, and AQP9 [13]. AQP1 is overexpressed in brain, breast, colorectal, and ovarian cancers [7]. AQP1 also displays higher levels of expression in lymph node metastasis than in their paired primary sites [24]. AQP3 is upregulated in cutaneous, esophageal and oral squamous, pulmonary, renal, or hepatocellular cancers [7]. AQP4 expression is increased in brain, lung, and thyroid cancers [7]. AQP5 is overexpressed in lung, chronic myelogenous leukemia, ovarian, stomach, pancreatic and colorectal cancers [7, 25, 26]. AQP7 displays increased expression in thyroid cancer [7]. AQP9 shows increased expression in ovarian cancer [7] and astrogliosis [27]. AQP3 is the most abundant skin AQP and the well-studied AQP in skin disorders and cutaneous biology (Figure 2) [28–32]. AQP3-knockout mice have impaired stratum corneum hydration compared with wild type mice [33]. Delayed wound healing processes with reduced keratinocyte proliferation and migration were also observed in AQP3 knockout mice [30, 31]. High expression of AQP3 in human squamous cell carcinoma and decreased skin tumorigenesis in AQP3 knockout mice has also been reported (Figure 2) [30, 31]. In addition to keratinocytes, AQP3 regulates human skin fibroblast migration, implying its role in wound healing [34]. The role of AQPs in various cancer types indicates a need for further evaluation to determine if they are suitable therapeutic targets.

Infectious (chronic viral, and bacterial) and parasitic diseases represent the third leading cause of cancer worldwide. Viruses are also known to utilize and regulate various aquaporin expression and functions in order to benefit from them for their successful life cycle. The AQP3 gene single-nucleotide polymorphism locus of aquaporin 3 (rs2231231) is associated with a susceptibility to Epstein-Barr virus (EBV)-associated nasopharyngeal carcinoma EBVaNPC and lymphoma EBVaL. The homozygous AA genotype is more frequently observed in individuals who develop EBVaNPC and EBVaL [35]. AQP4 antibody and concomitant herpes simplex virus 2 infection [36] has been associated with myeloradiculitis. A variety of neurological complications of Dengue virus infection such as dengue fever (DF) with the phenotype of neuromyelitis optica spectrum disorder (NMOSD) presented with brainstem symptoms or isolated unilateral optic neuritis tested positive for serum AQP4 antibody [37]. AQP4 antibodies are pathogenic and diagnostic for neuromyelitis optica spectrum disorder associated with multiple sclerosis (MS) [38]. Antibodies to AQP4 were also present in the serum of longitudinally extensive transverse myelitis (LETM) patient and plasma exchange was paralleled by disappearance of AQP4-Ab and sustained clinical improvement [39].

Human immunodeficiency virus (HIV) associated neurocognitive disorders (HAND) is associated with HIV neuropathy, HIV myelopathy, and HIV dementia. In order to study the astrocytic apoptosis in cortical degeneration and brain pathology, SIVmac239 and simian-human immunodeficiency virus (SHIV)-infected macaques and human AIDS autopsy cases were studied [40]. This study revealed vacuolar changes in perivascular processes of astrocytes, and expression of astrocyte-specific protein AQP4 by immunohistochemistry in SIVsm543-3 (biological isolate obtained late in AIDS rhesus macaques with SIV encephalitis) -infected macaques group, whereas APQ4 was diffusely positive in the neutrophil and perivascular area in control brains [40]. AQP4 levels were elevated in brain homogenates from the mid frontal gyrus of patients who died with HIV dementia (HIVD) [41].

Infection of human cells with Crimean-Congo hemorrhagic fever virus (CCHFV), an arthropod-borne pathogen strain IbAR 10200, downregulated AQP6 expression at the mRNA level. Interestingly, the overexpression of AQP6 in host cells decreased the infectivity of Hazara virus thus suggesting a protective role of AQP6 [42]. Rotavirus diarrhea, a major worldwide cause of infantile gastroenteritis, has also been reported to be linked to the downregulation of AQP1, AQP4, and AQP8 expression [43]. AQP3 has been reported as Kaposi’s sarcoma-associated herpes virus (KSHV) associated non-Hodgkin’s lymphoma (NHL) or primary effusion lymphoma (PEL) defining gene [44]. Involvement of AQP3 in PEL pathogenesis was studied in context with cyclooxygenase 2 (COX-2) inflammatory pathways, which is a target of nonsteroidal anti-inflammatory drugs (NSAIDs) [45]. AQP4 has been associated with intrinsic proinflammatory role in astrocyte swelling and cytokine release and a reduction in AQP4 water transport seems to be protective in neuroinflammatory CNS diseases such as autoimmune encephalomyelitis (EAE) [46].

## ROLE OF AQUAPORINS IN ONCOGENIC PATHWAYS

### Angiogenesis, tumor growth and metastasis

**Figure 3:**
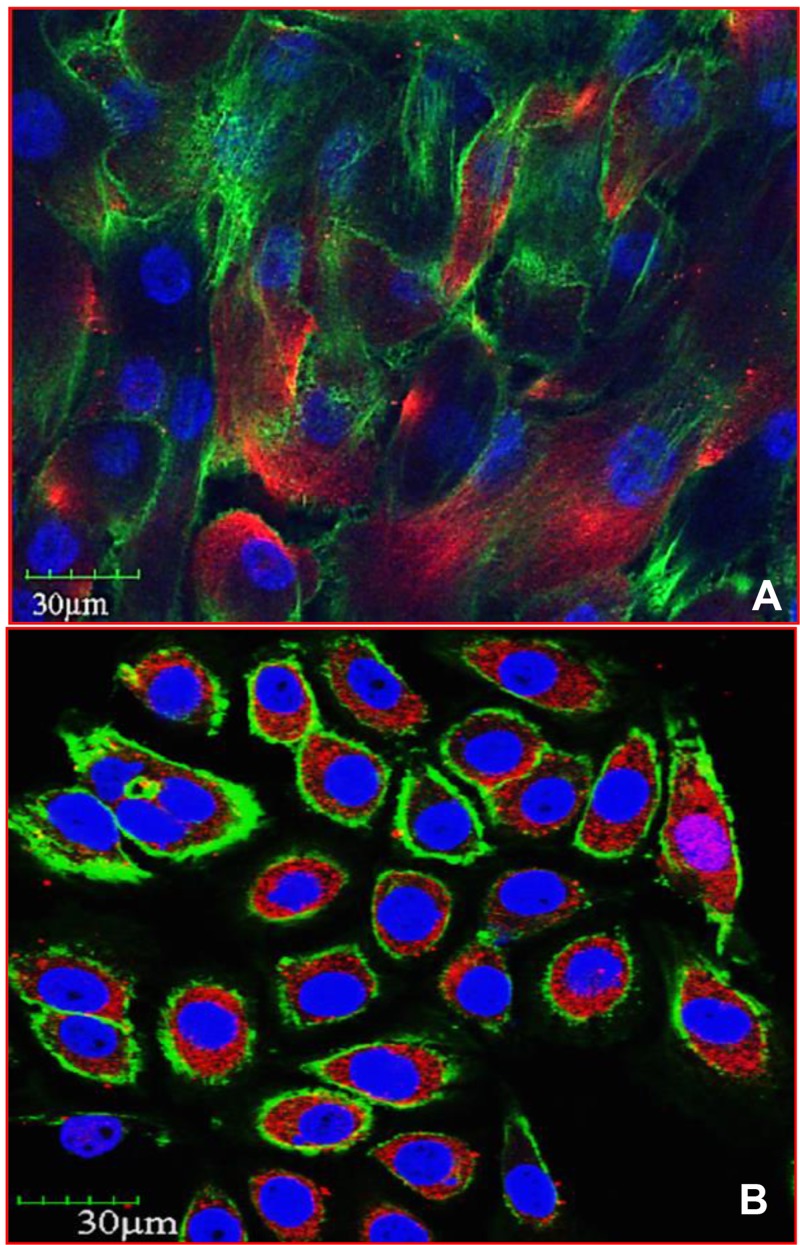
AQP3 expression is upregulated in inflammatory breast cancer (**A**) Human Mammary Epithelial Cells (HMEC; 830-05a, Cell Applications, San Diego, CA) and (**B**) Inflammatory Breast Cancer (149PT; Asterand, Detroit, MI) cell line were stained with anti-AQP3 antibody and analyzed by immunofluorescence and confocal microscopy staining for actin (green), DAPI (nuclei), and aquaporin (red). Magnification 60×.

AQPs -1,-2,-3,-4,-5,-8, and -9 are associated with cancer cell volume regulation, angiogenesis, cellular dissociation, migration, invasion, and metastasis [47]. Among AQPs, AQP1 plays a pivotal role in tumor angiogenesis and accelerates cell migration [7]. AQP1 is believed to play a role in the endothelial cell migration process that promotes tumor angiogenesis [9]. AQP3 plays a significant role in tumor biology as it alters cellular signaling; downstream protein expression patterns, encourages tumor development, and mediates cell migration. Uncontrolled cell proliferation and migration are considered hallmarks of cancer. While it is still unclear how AQPs facilitate cell migration, one proposed mechanism is that water entry into the leading edge of the cell enhances formation of the lamellapodium [48]. AQP3 is also implicated in skin tumor formation. AQP3 deficient keratinocytes display reduced glycerol and ATP content [49] and although there was reduced ATP content, there was no impairment of mitochondrial function. In further evaluation of AQP3 it has been shown that glycerol, imported via AQP3, serves as a regulator of cellular ATP energy and is necessary for lipid content development [49]. In summation, AQP3 is necessary for the development of lipid structures, by providing the necessary glycerol, and AQP3 is involved in cell proliferation and growth by encouraging ATP formation. AQP3 activity is further necessary for the expression of other downstream signaling proteins that play critical roles in tumor development and progression. We observed abundant expression of AQP3 in human IBC cell line SUM149PT [50–53] when compared to normal human mammary epithelial cells (HMEC) (Figure 3). Highly metastatic inflammatory breast cancer (IBC) is a rare and lethal form of breast cancer affecting roughly 1–6% of all breast cancer patients. IBC is treated using a multimodal approach but patients have a poor prognosis, and have a high mortality rate, due to the ineffective and toxic chemotherapy [54]. We observed similar results in MDA-IBC-3 cell line. The MDA-IBC-3 cell line was generated from primary human breast cancer cells isolated from pleural effusion fluid obtained from a patient with inflammatory breast cancer (IBC) at The University of Texas M. D. Anderson Cancer Center [55]. When AQP3 was experimentally knocked down in breast cancer cell lines, we noted a reduction in the active phosphorylated proteins extracellular signal-related kinase (ERK), protein kinase B (Akt), focal adhesion kinase (FAK), nuclear factor kappa light chain enhancer of activated B cells (NF-kB), and Src protein tyrosine kinase [our unpublished results]. ERK is a component in the well-known Ras-Raf pathway, which promotes the production of growth factors [56]. Akt has variable downstream actions that regulate cell growth, cell cycle progression, survival, migration, epithelial-mesenchymal transition, and angiogenesis [57]. FAK regulates cellular adhesion, motility, proliferation, and survival [58]. NF-kB is a critical link between inflammation and cancer. NF-kB blocks apoptosis in tumor cells, allowing for improper cell proliferation. Additionally, NF-kB assists in maintaining the tumor microenvironment and inflammatory response [59]. Src is noted to have a role in cancer cell metastasis, promoting the migration and proliferation of cancer cells [60]. These findings indicate that AQP3 expression plays a much larger role than simply transporting water. The numerous downstream effects and subsequent protein activation imparted by AQP3 further underline its importance in IBC progression. In lung cancer, overexpression of nuclear factor of activated T cells 5, also known as NFAT5, intimately interacts with AQP5, increasing AQP5 expression in order to promote cell proliferation and migration. These findings indicate that NFAT5 may play a role in the regulation of AQP5, thereby effecting osmoregulation in tumors [61]. Additional functions of other AQPs, particularly AQP0 and possibly AQP4 include facilitating cell-cell adhesion, and adhesion to the extracellular matrix which further renders therapeutic potential [62]. AQP5 has been suggested to perform more than simple water transport functions. AQP5 has been shown to interact with the Ras pathway in colon cancer, indicating that AQPs may play further roles in signaling than previously expected [63]. AQP5 has also been intimately linked with activation of the EGFR/ERK/p38 MAPK signaling pathway in lung cancer [64]. Developing a profound understanding of how AQPs are interacting with signaling pathways that are known to serve a critical role in cancer development and progression will be extremely important for the future development of therapeutic entities.

### Role of aquaporins in reactive oxygen species (ROS) and oxidative stress

**Figure 4:**
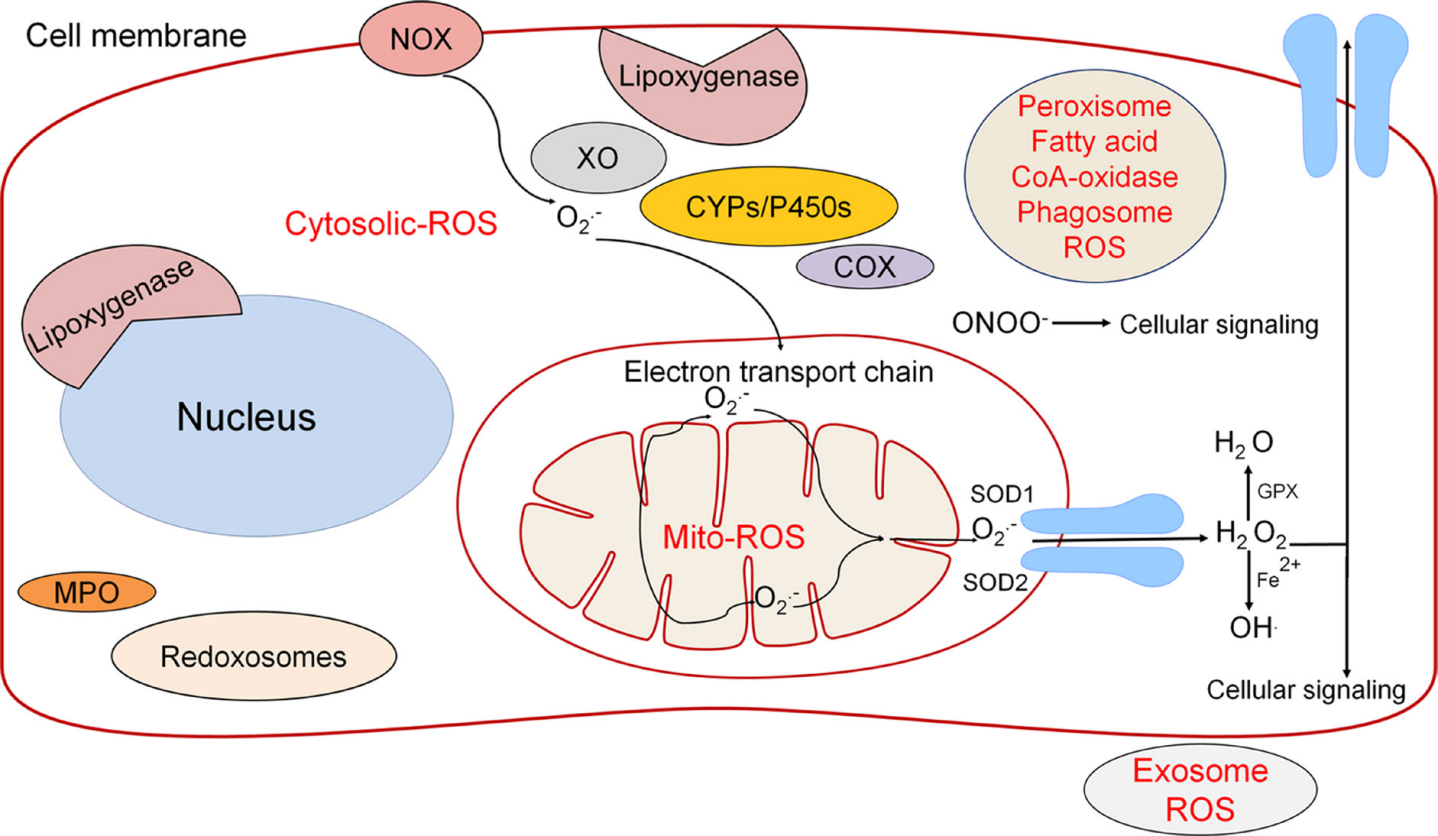
The source of ROS is regulated by several different pathways both in the cytoplasm and mitochondria Through the use of diffusion and AQPs, the transport of ROS molecules such as H_2_ O_2_ is utilized in downstream cellular signaling. ROS has historically been dismissed as an unwanted byproduct that leads directly to cell death; however, it has become clear that ROS plays a larger role in cellular signaling.

The role of ROS in cancer has been a growing area of research in recent years. Early investigators determined that ROS play an important role in damaging proteins, lipids and DNA, it should be therefore are believed to be tumorigenic by promoting genomic instability [65]. In more recent studies ROS species have been implicated as signaling molecules that regulate biochemical pathways thought to promote cancer. ROS is a common feature and a new possible target for screening in different cancers. Physiologically ROS are produced from xanthine oxidase (XO), myeloperoxidase (MPO), cyctochrome P450 (CYPs/P450s) [66], nicotinamide adenine dinucleotide phosphate oxidase (NOX), lipoxygenases (LO), and mitochondria (Figure 4) [67]. Cyclooxygenases (COXs; COX-1 and COX-2) are accountable for the formation of prostaglandins (PGs), which are involved in regulating inflammatory responses. COX-2 is inducible and is highly expressed in inflamed tissues and has been reported to induce ROS, which in turn can regulate COX-2 expression (Figure 4) [68]. Different biochemical pathways lead to cytoplasmic and mitochondrial ROS (mito-ROS) production (Figure 4). The biochemical synthesis of ROS such as peroxide, H_2_O_2_, and hydroxyl (OH) radicals are from superoxide. The mitochondrial electron transport chain is a main source of superoxide production: NADH-G oxidoreductase (Complex I) and Q-cytochrome c oxidoreductase (Complex III) [69, 70]. Other pathways that contribute to ROS include the NADPH oxidases (NOX family of oxidases), cytokine and growth factor receptors, and metabolic processes (Figure 4) [71, 72]. Recently, extracellular vesicles (EVs) or exosomes have been shown to contain functional NADPH oxidase 2 complexes and generate ROS [73].

ROS generators and scavengers play a balancing role in driving the proliferative state of the cell. Balancing the production of ROS in mammalians are antioxidants such as peroxiredoxins (PRXs), glutathione peroxidase (GPXs), and catalase [74–76]. PRXs are one of the most abundant proteins in the cell and contribute most to H_2_O_2_ degradation [75]. Accumulating evidences suggest that complex regulation of antioxidants facilitates control of specific ROS species levels leading to ROS mediated signaling events. For example, in response to activation of growth factor signaling, membrane-bound PRX1 can be phosphorylated to inhibit degradation of H_2_O_2_ [77]. Similarly, c-Abl and ARG (abl-related gene) tyrosine kinases can phosphorylate GPX1 causing increased activity, which protects against ROS [78]. In cancer the activation of nuclear factor (erythroid-derived 2)- like 2 (NRF2) increases the transcriptional response of antioxidant proteins [79]. Transcription factor NRF2 stabilization by inhibition of its negative regulator Kelch like ECH-associated protein 1 (KEAP-1) allows it to increase levels of antioxidants including GPXs and glutathione synthesis and utilization genes [80, 81]. NRF2 has also been illustrated to be important for tumorigenesis [82].

Of the multiple ROS signaling molecules, H_2_O_2_ and peroxynitrite (ONOO^-^) have been implicated to play an important role in cell signaling (Figure 4). Production of H_2_O_2_ is regulated by superoxide dismutases (SOD), such as SOD1-cytosolic, SOD2-mitochondrial, and SOD3-extracellular [83]. Following the formation of H_2_O_2_, hydroxyl radicals can be produced via Fenton chemistry (Fe2+ + H_2_O_2_→Fe3+ + OH**·** + OH−) [84]. ROS serve as signaling molecules to regulate biological processes, and have inherent chemical properties that confer reactivity to lipids, proteins, and DNA [85]. H_2_O_2_ is unique because it is stable and has specific reactions, but also depends on cellular context, local concertation, and exposure time [86]. H_2_O_2_ has been shown to be able to permeate cellular membranes yet there are different concentrations in different cellular compartments [67]. This discovery indicates that although H_2_O_2_ can permeate cellular membrane it is still diffusion limited and therefore contributes to the validity of it being an important form of cellular signaling [87]. Through different regulatory mechanisms H_2_O_2_ has been found to be used for hypoxic signal transduction, cell differentiation, cell proliferation, and mediation of immune responses [88]. Different concentrations of H_2_O_2_ can lead to modification of susceptible residues on target molecules such as oxidation of cysteine residues on proteins [89]. Another example is inactivation of phosphatases via H_2_O_2_ allows growth factor dependent signaling. For example, vascular endothelial growth factor (VEGF) and angiopoietin-1, lead to the induction of genes involved in angiogenesis and thus represent therapeutic targets to inhibit angiogenesis. H_2_O_2_ is an important regulatory molecule utilized in vital biochemical pathways and not an unwanted or rather toxic byproduct. Similarly, peroxynitrite ONOO- is a regulatory ROS that affects downstream pathways including NFkB, EGFR, PDGFR, α and β-adrenoreceptors, Src, JNK, ERK, p38, PI3K, PKB, and PKC. Reactive nitrogen species (RNS) compared to H_2_O_2_ is likely to have overlapping and distinct roles in signaling. The mitochondria play a role in regulating signaling pathways via H_2_O_2_ through three main pathways, phosphoinositide 3-kinase (PI3K) signaling, hypoxia-inducible factors and metabolic adaptation.

Because there are fluctuating concentrations of ROS in different compartments of the cell, the regulation of permeability is important to control signaling. The permeability of H_2_O_2_ is controlled by AQPs that contribute to the gradient. Mammals have thirteen different AQPs that regulate the flow of small-uncharged molecules like ammonia, CO2, glycerol, urea, H_2_O_2_ and water [90]. AQPs that are able to transport considerable amounts of H_2_O_2_ are AQP1, AQP3, AQP5, and AQP9 denoted peroxiporins [91]. AQP3 has been shown to be expressed by epithelial cells where change in expression is observed in response to inflammation [92]. AQP8 has been speculated to play a role in regulating the mitochondrial transport of H_2_O_2_ out of the mitochondria-one of the largest sources of ROS- as illustrated in reconstituted yeast and transfected mammalian cells [93, 94]. Besides promoting water and glycerol transport, AQP3 is also indicated in H_2_O_2_ transport. When AQP3 is knocked down, transport of extracellular H_2_O_2_ into cells decreased, consequently leading to diminished tumor growth [95]. These findings have led to the proposition that the increased intracellular H_2_O_2_ levels act as a second messenger during epidermal growth factor receptor signaling, leading to tumor progression [95]. AQP3 mediated H_2_O_2_ has been described as controlling EGF signaling in epithelial cells and plays an important role in T-cell and breast cancer cell migration [11, 95, 96]. The importance of AQPs has been a point of research as increasing evidence indicates it is a key transporter for many cancers. This is illustrated by the finding that AQP3 is required for C-X-C motif chemokine 12 (CXCL12)-induced breast cancer cell signaling and directional migration through a mechanism in which CLCL12-induces the formation of extracellular H_2_O_2_ and subsequent internalization. AQP3 knockdown cells consistently showed markedly reduced breast cancer cell metastasis to lungs. AQP8 has been shown to transport NADPH-oxidase 2 (NOX2) generated H_2_O_2_ across the plasma membrane to promote B cell signaling and efficient induction of B cell activation and differentiation [97]. AQP8 is able to modulate NOX derived H_2_O_2_ transport through the plasma membrane affecting redox signaling linked to acute leukemia cell proliferation [98]. Colonic epithelial cells produce extracellular H_2_O_2_, which acts as a potent signaling molecule and signal transduction by H_2_O_2_ depends on entry into the cell by transit through AQP3 [99]. In response to injury, AQP3-depleted colonic epithelial cells showed defective lamellipodia, focal adhesions, and repair after wounding, along with impaired H_2_O_2_ responses after exposure to the intestinal pathogen. AQP3-depleted colonic epithelial cells had reduced epithelial expression of IL-6 and TNF-α, and impaired bacterial clearance elucidating the signaling mechanism of extracellular H_2_O_2_ and also implicates the role of AQP3 in innate immunity at mucosal surfaces [99]. AQP3 facilitated transport of H_2_O_2_ has been reported to regulate T-cell migration towards chemokines, which subsequently stimulates Rho-GTPase signaling [100]. Extracellular H_2_O_2_ transport in colonic epithelium has been reported to be AQP3 mediated, and has role in innate immune responses at mucosal surfaces since mice lacking AQP3 had impaired healing of the superficial colon [100]. Helicobacter pylori (H. pylori), the major stomach carcinogen, promotes AQP3 expression via the ROS-HIF1α-AQP3-ROS axis in stomach mucosa [101]. This loop presents a potential novel mechanism for cancer pathogenesis in gastric cancers. H. pylori infection also stimulates the AQP3 level dependent production of proinflammatory cytokines IL-6, IL-8, and TNFα, which adds up to the progression of gastric carcinomas [101]. Oxidative stress induces an increase in the membrane expression of AQP4 in astrocytes and is regulated by caveolin-1 phosphorylation [102]. The oxidative stress induced increase in the membrane expression of AQP4 was inhibited by the antioxidant N-acetylcysteine (NAC) [102].

## AQPS AS BIOMARKERS

In hepatic cancer, immunohistochemical detection of AQP1 is reliable for differentiating between cholangiocarcinoma, hepatocellular carcinoma, and metastatic colorectal carcinomas [7]. Furthermore, measuring AQP1 levels in the urine has proven to be a useful biomarker for renal cell carcinoma. Subjects with known renal cell carcinoma have a 12-fold increase in urine AQP1 concentration [103]. A reduced progression-free and overall survival for the AQP1 positive cutaneous melanoma patients has been reported [104]. AQP1 expression has also been associated with an adverse prognosis in cutaneous melanoma [104].

In adenoid cystic carcinoma it has been noted that AQP1 promoter hypomethylation is common, additionally, AQP1 tends to be overexpressed. Although AQP1 is overexpressed, its expression levels are not associated with outcomes. However, increased AQP1 methylation is associated with improved prognosis and may serve as a useful prognostic marker for future evaluation [105]. AQP3 has proven to serve as a potential prognostic marker after curative surgery in early breast cancer demonstrating human epidermal growth factor receptor 2 (HER2/neu, c-erbB2) overexpression [106]. HER2 is a membrane tyrosine kinase and oncogene, when activated it provides the cell with potent proliferative and anti-apoptosis signals, and confers aggressiveness to breast cancers [106]. AQP3 and AQP5 overexpression indicates a poor prognosis and low 5-year survival rates for patients with hepatocellular carcinoma. AQP5 overexpression has been observed in lung adenocarcinoma and colorectal cancer; therefore, it is a prognostic biomarker. In a sampling of 45 colorectal cancer tumor specimens, AQP5 demonstrated high levels of expression in 14 samples, moderate levels of expression in 29 samples, and absence of expression in 2 samples. Increased expression levels were correlative to TNM stage (classification of malignant tumors), lymph node metastasis, and distant metastasis indicating that AQP5 expression level may serve as prognostic marker. Additionally, patients with an absence of AQP5 expression had a greater cumulative survival rate [107]. AQP5 may also serve as a prognostic marker for ductal breast cancer. Decreased AQP9 expression may indicate resistance to apoptotic stimulation in hepatocellular carcinoma [7]. AQPs play very important roles in intercellular communication via their expression on EVs or exosomes [108]. EVs include exosomes (30–150 nm) derived from so called multivesicular endosomes or multivesicular bodies (MVBs), microvesicles (50–2000 nm), and apoptotic bodies (500–4000 nm). AQP1 and AQP2 have been found in urinary EVs and the levels of urinary exosomal AQP1 and AQP2 has been correlated with their renal protein levels and detection of renal injury [108]. AQP1 sorting into exosomes has been observed during *in vivo* physiologic process of erythropoiesis/reticulocyte maturation [109]. AQP5 has been detected in salivary exosome secretions and is correlated to patients with diabetes mellitus or Sjögren's syndrome. Salivary AQP5 has been proposed as a valuable biomarker for diagnosis of dry mouth or xerostomia. These findings indicate the strong potential of AQP expression serving as a suitable clinical biomarker.

## THERAPEUTIC POTENTIAL OF AQPS

AQP5 over-expression is associated with poor prognosis of patients, and the cancers with high AQP5 exhibit EMT and activation of signaling cascades such as EGFR/ERK/p38 MAPK pathway. Another interesting aspect of AQP5 overexpressing tumors is phosphorylated cAMP-protein kinase (PKA) consensus site in AQP5 that favors cell proliferation [19]. Ser156 in AQP5 is a potential therapeutic target due to its role in lung cancer cell proliferation and invasion [19]. Additionally, developing a monoclonal antibody is another potential approach [7]. Downregulation of AQP1 may be a potential therapeutic goal to minimize cell migration for colorectal cancer patients [7]. AQP3 plays a role in tumor differentiation and metastasis along with epidermal growth factor receptors in colorectal cancer, and therefore, these both may be potential targets for inhibition [7]. AQP3 inhibition by siRNA increased prostate cancer cells’ sensitivity to cryotherapy [110, 111]. The inhibition of AQP3 by RNA interference retarded the growth and invasiveness of XWLC-05 lung cancer cells and decreased the activity of matrix metalloprotease-2 (MMP2) [112]. AQP3 overexpression in gastric cancer cells facilitates cisplatin resistance via autophagy suggesting that the development of AQP3 based tumor therapeutics could play a key role in future gastric cancer treatment strategies [113]. AQP5 and AQP9 are potential targets to increase the effectiveness of chemotherapeutic drugs among colorectal cancer patients. AQP9 is a potential target for hepatocellular carcinoma due to its decreased expression, which reduces apoptotic stimulation [7]. Additionally, AQP9 is a therapeutic target for brain cancer due to its role in astrocytic glioma. AQP permeability for anions under certain conditions, or after induction of a single point mutation, may serve as a unique option for therapeutic application [4]. Particular precautions must be taken when developing drugs based on AQPs due to the ubiquitous action of AQPs. Loss of function in AQPs may cause congenital cataracts (AQP0), diabetes insipidus (AQP2), and autoantibodies against AQP4 cause the autoimmune demyelinating disease neuromyelitis optica [114]. Previously manufactured drugs such as Bevacizumab and Ranibizumab target VEGF, combining this therapy with an AQP1 inhibitor may allow for further disruption of tumor angiogenesis [9]. A unique way to target AQP1 may be through manipulation of osmolarity. In previous experiments, cells that were not stimulated by constant changes in osmolarity may selectively downregulate AQP1, while conversely, AQP1 was upregulated by hypertonic challenge in cells lacking endogenous expression [9]. AQPs have been suggested to be a novel therapeutic target for anti-inflammatory therapy in a model of acute lung inflammation. AQP blockade or deficiency reduced IL-1β release by NLRP3 activators stimulated macrophages suggesting the involvement of NLRP3 inflammasome axis [115]. The overwhelming numbers of processes that AQPs participate in illustrate their value in clinical medicine. AQPs are ubiquitously expressed and implicated in dozens of cancer types (Figure 2). The varying levels of AQP expression in different cancer types may be exploited as diagnostic and prognostic markers. Having additional methods of detection may lead to earlier detection of cancer, which very well may make a major impact on patient outcomes. Certain cancer types are particularly difficult to detect, one example being lung cancer. If measurements of AQP expression, particularly AQP5, which is overexpressed in lung cancer, may be reliably used as a diagnostic marker, we may be able to detect lung cancer at much earlier stages. Additionally, if AQP expression may be utilized as a prognostic marker, clinicians may be able to give patients more accurate expectations in terms of outcomes. Finally, if AQPs can be safely targeted and expression can be manipulated, this may provide a novel mechanism of chemotherapy.

## PERSPECTIVES

The results acquired from studies thus far indicate that there are many possible potential therapies involving AQPs, such as AQPs targeted inhibitors, AQPs-specific monoclonal antibody, or AQP gene transfer [7]. Understanding AQP function and gating properties is essential in order to understand the AQP contribution to homeostasis and cellular environments. This is particularly clinically valuable as manipulating AQP function may offer therapeutic advantages. Moreover, the role of aquaglyceroporin mediated glycerol transport in cell proliferation and adipocyte metabolism and its role in cancer progression point to the aquaglycerolporin subfamily as potential therapeutic targets [2]. AQPs, particularly AQP4, are not only implicated in cancer, they also play a role in the development and progression of demyelinating diseases [116] such as neuromyelitis optica, and metabolic pathologies such as obesity, dermatological pathologies, edema, and glaucoma [117]. Since AQPs play such a pivotal role in glycerol transport they are consequently implicated in lipid balance disturbances and may play into the development of obesity and diabetes [118]. For obesity and metabolic pathologies AQP7 modulation therapy may be a viable solution to combat this ever-growing epidemic [118]. In the cardiovascular system AQP1, AQP4, AQP7, and AQP9 are expressed in endothelial cells, vascular smooth muscle cells, and cardiac tissue which all play a vital role in the development and progression of pathologies such congestive heart failure, ischemia, hypertension, and angiogenesis [119]. Loss of function mutations in human AQP0 cause congenital cataracts and AQP2 causes nephrogenic diabetes insipidus. Autoantibodies against AQP4 cause the autoimmune demyelinating disease neuromyelitis optica [114]. Identification of AQPs on urinary and salivary exosomes has significant implications for the development of biomarkers for renal and oral disease diagnosis. Although there are potential AQP modulators identified and tested but there are a lot of challenges accompanying with the development of better modulators, which include the ability to target with specificity [114]. Furthering our understanding of AQPs is necessary to potentially develop novel therapeutics against some of our greatest public health threats as cancer, cardiovascular disease, neurological pathology, and obesity.

## Abbreviations

AQP: Aquaporin
HIF: Hypoxia-Inducible Factor
H_2_O_2_: Hydrogen Peroxide
HMEC: Human Mammary Epithelial Cells
ERK: Extracellular signal-related kinases
Akt: Protein Kinase B
FAK: Focal Adhesion Kinase
NF-kB: Nuclear Factor Kappa Light Chain Enhance of Activated B Cells
SRC: Proto-oncogene tyrosine-protein kinase SRC
EGFR: Epidermal Growth Factor Receptor
NFAT5: Nuclear Factor of Activated T-cells 5
IBC: Inflammatory Breast Cancer
ER: Estrogen Receptor
HER2: Human Epidermal Growth Factor Receptor
MAPK: Mitogen Activated Protein Kinase
NOX: Nicotinamide Adenine Dinucleotide Phosphate Oxidase
COX-2: Cyclooxygenase-2
EV: Extracellular Vesicle
MMP2: Matrix Metalloprotease-2
MVB: Multivesicular Body
ONOO^−^: Peroxynitrite
SOD: Superoxide Dismutase
EMT: Epithelial Mesenchymal Transition
VEGF: Vascular Endothelial Growth Factor
FGF-2: Fibroblast Growth Factor-2
PRX: Peroxiredoxin
GPX: Glutathione Peroxidase
RNS: Reactive Nitrogen Species
XO: Xanthine Oxidase
LO: Lipoxygenase
ROS: Reactive Oxygen Species
EAE: Autoimmune Encephalomyelitis
EBV: Epstein - Barr virus
NPC: Nasopharyngeal Carcinoma
NRF2: Nuclear Factor (erythroid-derived 2)- like 2
TNBC: Triple Negative Breast Cancer
KSHV: Kaposi’s sarcoma Associated Herpes Virus
NHL: Non-Hodgkin’s Lymphoma
PEL: Primary Effusion Lymphoma
